# Impact of the Novel Coronavirus 2019 (COVID-19) Pandemic on Head and Neck Cancer Care

**DOI:** 10.1177/01945998211004544

**Published:** 2022-01

**Authors:** Joshua Adam Thompson, Joshua E. Lubek, Neha Amin, Reju Joy, Donita Dyalram, Robert A. Ord, Rodney J. Taylor, Jeffrey S. Wolf, Ranee Mehra, Kevin J. Cullen, Jason K. Molitoris, Matthew Witek, John C. Papadimitriou, Robert E. Morales, Kyle M. Hatten

**Affiliations:** 1 Department of Otorhinolaryngology, University of Maryland School of Medicine, Baltimore, Maryland, USA; 2 Department of Oral and Maxillofacial Surgery, University of Maryland, Baltimore, Maryland, USA; 3 University of Maryland School of Medicine, Baltimore, Maryland, USA; 4 University of Maryland Greenebaum Comprehensive Cancer Center, Baltimore, Maryland, USA; 5 Department of Radiation Oncology, University of Maryland School of Medicine, Baltimore, Maryland, USA; 6 Department of Pathology, University of Maryland School of Medicine, Baltimore, Maryland, USA; 7 Department of Diagnostic Radiology, University of Maryland School of Medicine, Baltimore, Maryland, USA

**Keywords:** coronavirus, COVID-19, head and neck cancer, tumor conference

## Abstract

**Objective:**

The study aimed to assess the impact of the coronavirus disease 2019 (COVID-19) pandemic on head and neck oncologic care at a tertiary care facility.

**Study Design:**

This was a cross-sectional study conducted between March 18, 2020, and May 20, 2020. The primary planned outcome was the rate of treatment modifications during the study period. Secondary outcome measures were tumor conference volume, operative volume, and outpatient patient procedure and clinic volumes.

**Setting:**

This single-center study was conducted at a tertiary care academic hospital in a large metropolitan area.

**Methods:**

The study included a consecutive sample of adult subjects who were presented at a head and neck interdepartmental tumor conference during the study period. Patients were compared to historical controls based on review of operative data, outpatient procedures, and clinic volumes.

**Results:**

In total, 117 patients were presented during the review period in 2020, compared to 69 in 2019. There was an 8.4% treatment modification rate among cases presented at the tumor conference. There was a 61.3% (347 from 898) reduction in outpatient clinic visits and a 63.4% (84 from 230) reduction in procedural volume compared to the prior year. Similarly, the operative volume decreased by 27.0% (224 from 307) compared to the previous year.

**Conclusion:**

Restrictions related to the COVID-19 pandemic resulted in limited treatment modifications. Transition to virtual tumor board format observed an increase in case presentations. While there were reductions in operative volume, there was a larger proportion of surgical cases for malignancy, reflecting the prioritization of oncologic care during the pandemic.

The severe acute respiratory syndrome coronavirus 2 (SARS-CoV-2) has resulted in a global pandemic attributable to the widespread transmission of the coronavirus disease 2019 (COVID-19). Infected individuals experience a wide range of presentations from an asymptomatic carrier to acute respiratory distress syndrome. The virus primarily infects the nasal and bronchial epithelium with an average incubation period of 5 days, although symptoms may still arise 14 days after infection.^1,2^ Transmission via respiratory droplets and aerosolization has occurred rapidly, especially given that presymptomatic carriers are responsible for an estimated 48% to 62% of transmission.^3^

The COVID-19 pandemic has drastically affected cancer treatment paradigms as it has introduced multilevel risk for the patient and provider in the management of head and neck cancer (HNC). Oncology patients are considered a high-risk population due to preexisting medical comorbidities and treatment regimens that can result in immunosuppression and postsurgical intensive care needs requiring complex respiratory care.^4,5^ Moreover, otolaryngologists, oral-maxillofacial surgeons, and other head and neck health care providers face increased risks of exposure given that diagnostic endoscopies and operative procedures of the upper aerodigestive tract are aerosol-generating procedures.^6-8^ In an effort to minimize both patient and provider exposure risks, the American College of Surgeons and the American Academy of Otolaryngology–Head and Neck Surgery recommended delaying elective surgical cases or choosing nonsurgical management where there would not be an impact on patient outcomes.^6,9-12^ In addition, 42 states implemented stay-at-home orders to limit the public’s exposure to COVID-19, while the Maryland Department of Health released an additional order prohibiting all elective and nonurgent medical procedures effective March 24, 2020, until May 11, 2020.^13^ Thus, as statewide restrictions and various risk stratification protocols continue to recommend modifications in clinical management, it has become important to assess the implementation of these guidelines on patient care.

Overall, the impact of operative prioritization and recommendations remains largely unknown for patients and providers apart from survey data. In one such study involving 88 head and neck surgeons, the majority favored delaying treatment up to 4 weeks for early stage oral cavity and glottic cancer.^14^ Surgeons demonstrated more willingness to delay care. However, the final treatment decisions and their rationale have yet to be assessed in outcomes-based research.

The aim of this study is to assess the impact on access to oncologic services as well as the treatment modifications made by the University of Maryland Medical Center (UMMC) Head and Neck Interdisciplinary Tumor Board. The study will examine trends in oncologic management based on historical data. We hypothesized that the COVID-19 pandemic would lead to treatment modifications, which are intended to reduce the risk of SARS-CoV-2 exposure and lead to alteration in treatment modalities.

## Methods

This study used a prospective observational cohort design with a comparison to historical data. Our study was submitted to the University of Maryland, Baltimore, Institutional Review Board (IRB) and was granted IRB exemption. Patients over 18 years of age who presented for head and neck oncologic care at the University of Maryland Medical Center were followed at their initial consultation and treatment. Patients were identified during a multidisciplinary tumor board (MDTB) conference, which includes representatives from otolaryngology–head and neck surgery, oral maxillofacial surgery, radiation oncology, and medical oncology. Data collection occurred during institutional and statewide restrictions on elective surgery and outpatient clinic visits.

Impacts of the COVID-19 pandemic were identified and categorized from a multi-item flowchart that was drafted and approved by members of the MDTB. Treatment modifications were classified as follows: elimination of systemic therapy, treatment delay, change to nonsurgical management, or alteration in adjuvant therapy. The rationales of any modifications were identified as 1 or more of the following categories: operating room limitations, medical comorbidities, COVID-19 positive, patient concerns, or social limitations. Operating room limitations included lack of appropriate personal protective equipment or reductions in operating room availability. Social limitations included patient-related factors such as travel restrictions, lack of family support, decreased access to transportation services, or reduced access to primary care providers.

### Collection of Tumor Conference Information

Information regarding treatment modifications was collected prospectively during weekly MDTB conferences from March 18, 2020, to May 20, 2020. The presence of a modification, type of modification, and rationale for modification were discussed and recorded for each patient presented. Nearly all patients who present to clinic or who undergo a procedure for treatment or diagnosis are presented at the MDTB. If a patient was presented more than 1 week, the initial presentation was counted toward the volume of cases presented. Distinction was made between initial cancer consultations and presentations of patients under cancer surveillance. Tumor and patient characteristics were obtained from a combination of tumor conference review and chart review. As a historical control, information regarding the number of new and total case presentations at the tumor conference during the same 2-month time period in 2019 were obtained from a Research Electronic Data Capture (REDCAP) database. As a supplement to tumor conference data, deidentified metrics of outpatient clinic volumes, procedural data, and surgical cases were obtained from electronic medical records during the study period and compared to 2019. Outpatient clinic volumes, procedural data, and surgical cases included those under the care of the same 6 head and neck surgeons within the Department of Otorhinolaryngology and Department of Oral Maxillofacial Surgery who practiced during 2019 and 2020.

Statistical analysis was conducted with GraphPad Prism (GraphPad Software). Observed and expected comparisons were made between the 2019 cohort of patients and the 2020 cohort of patients. In addition, patient and tumor demographics were compared between the annual cohorts as well as between those patients whose treatment plans were modified and those whose treatment plans were unmodified. Chi-square and Fisher exact tests were used where appropriate to make comparisons between the groups with a level of significance of *P* < .05.

## Results

**Figure 1.**
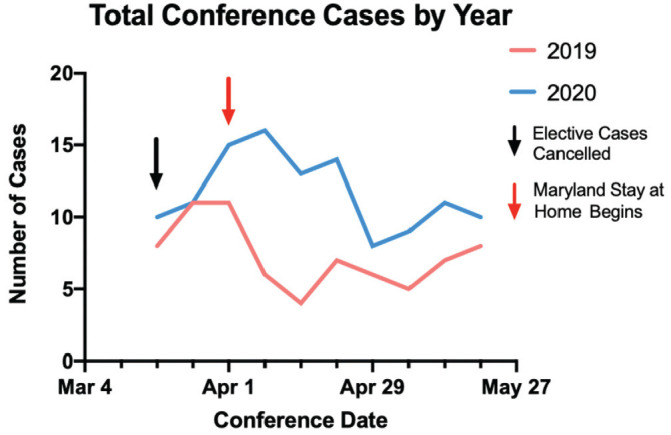
Multidisciplinary tumor board presentations (MDTB) by year. Total cases presented in the MDTB over the review period in 2020 and in 2019. Arrows designate date of implementation of institutional and statewide policies.

In total, 117 patients were presented for oncologic care and case discussion at the weekly tumor conference during the review period in 2020 via virtual tumor board web-based meetings. During the same period of time in 2019, there were 69 patients presented during in-person meetings. In 2020, 66% of patients were male, with the most common site of malignancy being the oral cavity. In 2019, 74% of patients were male, with the most common site being the oropharynx. Other reported primary sites included cutaneous malignancies, laryngeal malignancies, and sinonasal malignancies. In 2019 and 2020, there was a greater proportion of early tumor (T1 or T2) stage and early nodal (N0 or N1) stage compared to more advanced disease (**Table 1**). There were more total and new cancer MDTB case presentations in 2020 than in 2019. While the volume of surgical cases presented decreased during the review period, this was similar to the previous year (**Figure 1**).

**Table 1.** Characteristics of Patients Presented at the Multidisciplinary Tumor Board.

Characteristic	2019 (N = 69)	2020 (N = 117)	*P* value
Age, mean, y	65.2	63.5	NS
Sex, No.			NS
Male	51	77	
Female	18	40	
Primary site, No.			.04
Sinonasal	5	4	
Salivary gland	0	8	
Cutaneous	9	11	
Oral cavity	16	52	
Oropharynx	24	25	
Nasopharynx	0	2	
Larynx	8	10	
Other	6	5	
T stage, No.			NS
1	13	28	
2	16	27	
3	12	13	
4	9	18	
N stage, No.			NS
0	28	41	
1	8	17	
2	9	15	
3	2	4	

Abbreviation: NS, not significant.

**Figure 2.**
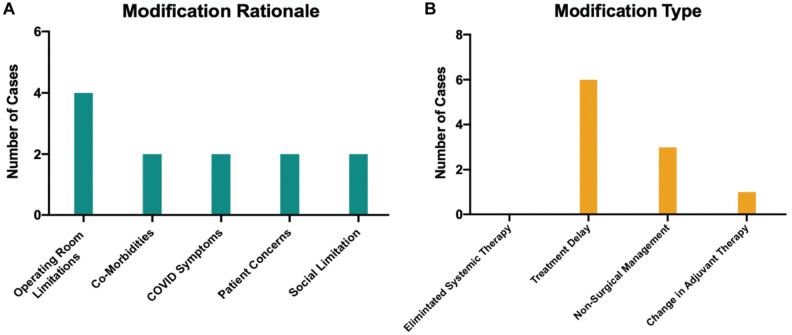
Modification rationales and types. (a) Frequency with which various rationales were cited for treatment modifications. Multiple rationales could be cited for a single patient. (b) Frequency of the types of modifications in patients presented at the multidisciplinary tumor board.

**Figure 3.**
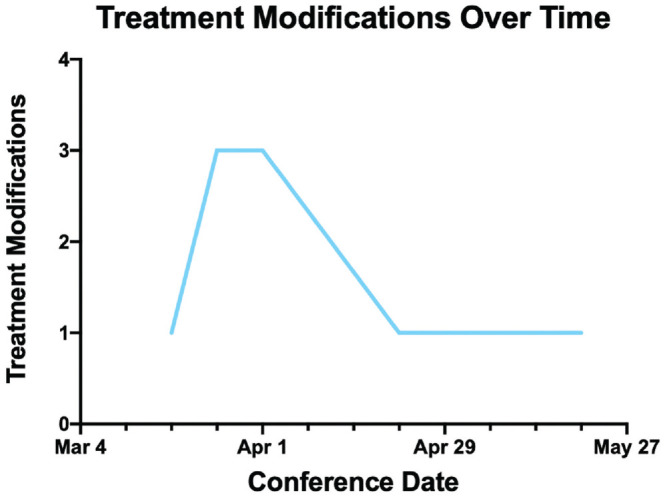
Frequency of treatment modifications in multidisciplinary tumor board patients over the review period.

The frequencies of modifications and the rationales for modifications were recorded prospectively. Of the 117 patients presented in the MDTB, 10 (8.4%) treatment modifications were attributed to the COVID-19 impact. There were no statistical differences in baseline characteristics between the patients with modifications and those without modifications (**Table 2**). The rationales for treatment modification and types of modifications are shown in **Figure 2**. The most common type of modification was a treatment delay, while the second most common modification was a change from primary surgical management to nonsurgical management. The most common reason for modification was operating room limitations, which was reported in 4 of 10 patients. Treatment modifications tended to occur earlier in the course of this institutional response to the pandemic, as seen in **Figure 3**. The characteristics of the 10 patients with treatment modifications are presented in **Table 3**.

**Table 2.** Characteristics of Multidisciplinary Tumor Board Patients With and Without Modifications.

Tumor conference characteristics of modified and unmodified cases				
Category	All patients (N = 117)	Unmodified (n = 107)	Modified (n = 10)	*P* value
Age, mean, y	63.6	63.4	76.8	NS
Sex, No.				NS
Male	77	70	7	
Female	40	37	3	
New cancer, No.	78	72	6	NS
Existing cancer, No.	39	35	4	
Cancer site, No.				NS
Sinonasal	4	3	1	
Salivary	8	7	1	
Cutaneous	11	11	0	
Oral cavity	52	48	4	
Oropharynx	25	22	3	
Nasopharynx	2	2	0	
Larynx	10	10	0	
Other	5	4	1	
T stage, No.				NS
1	28	26	2	
2	27	26	1	
3	13	13	0	
4	18	13	5	
N stage, No.				NS
0	41	39	2	
1	17	13	4	
2	15	15	0	
3	4	4	0	

Abbreviation: NS, not significant.

**Table 3.** Characteristics of Multidisciplinary Tumor Board Patients With Treatment Modifications.

Characteristics of patients with treatment modifications										
Sex	Tumor site	Subsite	Path	T	N	M	Prior therapy	Tumor board recommendation	Treatment modification	Reason(s) for modification
M	CaUP	NA	SCC	x	1	x	None	TORS CaUP/neck	Treatment delay	Patient concern
M	Oropharynx	Tonsil	SCC	1	1	0	None	Surgical excision, adjuvant XRT	Nonsurgical management	Operating room safety, systems limitations
M	Oral cavity	Hard palate	SCC	4b	x	x	Surgery	Adjuvant CXRT	Alteration in adjuvant therapy	Medical comorbidities
M	Oropharynx	Base of tongue	SCC	1	1	0	None	TORS	Nonsurgical management	Operating room safety, systems limitations
F	Sinonasal	Left orbit	ACC	4a	x	x	CXRT, surgery	Operative biopsy	Treatment delay	Operating room safety
F	Parotid	Left	AGS	4a	0	0	Surgery, XRT	Surgical excision with or without reirradiation	Nonsurgical management	Medical comorbidities
M	Oral cavity	Mandible	ORN	—	—	—	None	Surgical excision	Treatment delay	Patient concern
F	Oral cavity	RMT	SCC	2	0	x	None	Surgical excision	Treatment delay	COVID-19 symptoms
M	Oral cavity	Oral tongue	SCC	4	1	x	None	Surgical excision	Treatment delay	COVID-19 positive
M	Oropharynx	Base of tongue	SCC	4a	0	0	CXRT	Surgical excision	Treatment delay	Operating room safety

Abbreviations: ACC, adenoid cystic carcinoma; AGS, angiosarcoma; CaUP, carcinoma unknown primary; COVID-19, coronavirus disease 2019; CXRT, chemoradiation; NA, not applicable; ORN, osteoradionecrosis; RMT, retromolar trigone; SCC, squamous cell carcinoma; TORS, transoral robotic surgery; XRT, radiation; —, not applicable.

**Figure 4.**
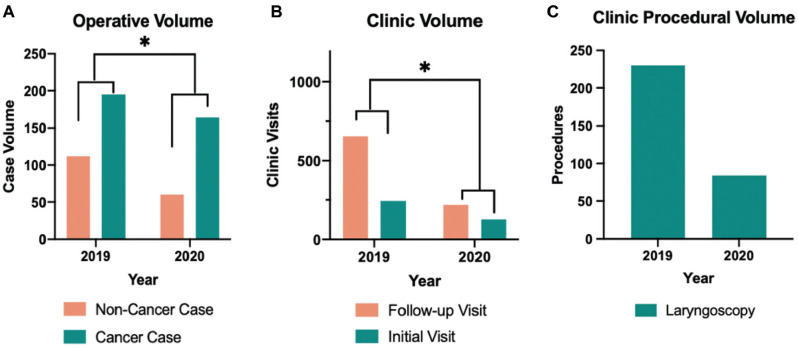
Changes in outpatient clinical and procedural volumes. (a) Operative volumes over a 4-month period. (b) Outpatient clinic visits over the same time periods. (c) Flexible laryngoscopies performed in 2019 and 2020. *P < .05.

The outpatient clinic and operating room case volumes were retrospectively analyzed during the restriction compared to historical comparisons from 2019. In 2020, there were significantly fewer operating room cases, 224, compared to 307 in 2019 (*P* = .02). In addition, the outpatient setting observed a significant reduction in office visits in 2020, 346 encounters, compared to 2019, 898 encounters (*P* < .001). However, there was a greater proportion of cancer surgeries (73% vs 64%) and initial patient visits (37% vs 27%) in 2020 compared to 2019 (**Figure 4a,b**). The number of outpatient laryngoscopies performed decreased by 63% from 2019 to 2020 (**Figure 4c**).

## Discussion

The purpose of this study was to assess the impact of the COVID-19 pandemic on care for head and neck oncologic patients compared to historical controls. Virtual meeting formats allowed for weekly meetings of the MDTB conference, which recorded an increase in the number of patients reviewed compared to the prior year. Overall, there were relatively few modifications made to treatment plans, which were most commonly a treatment delay. The delays were not recommended during the MDTB, but unanticipated events due to COVID testing and operating room limitations. Treatment modifications were also not associated with a particular tumor primary site, tumor stage, or patient demographic. While outpatient and operative volumes decreased during the pandemic compared to the prior year, the proportion of oncologic cases and the proportion of new patient visits were significantly greater during the pandemic. This reflected the prioritization and triage of oncologic patients at this institution during the response to the pandemic.

The ongoing COVID-19 pandemic resulted in restrictions and prioritization of medical care in an effort to reduce patient and health care exposure. Statewide travel and health care restrictions were first introduced by the state of Washington to prioritize emergent and life-threatening health conditions.^15^ Similarly, the state of Maryland and the University of Maryland Medical System implemented policies to limit the spread of the virus, which included a hold on elective procedures and outpatient visits on March 18, 2020. At the time of the restrictions, statewide reporting of respiratory specimen testing for SARS-CoV-2 was 11.3% and later peaked at 26.9% on April 17, 2020. Following the virus peak, there was a gradual decline in SARS-CoV-2 testing positivity, which led to a lifting of restrictions and resumption of elective procedures in June 2020 at UMMC.^16^

Quantifiable evidence of the pandemic’s impact on access to oncologic care and treatment of these patients during government-implemented restrictions remains limited. The University of Washington proposed continuing definitive oncologic care for solid tumors despite infectious risks, but the authors acknowledged that complications during therapy may arise and further stress clinical resources.^15^ In addition, Weinstein et al^17^ published a consensus recommendation regarding suggesting changes in practice management for patients with head and neck cancer, in which they recommended prioritization of standard of care therapy. While adherence to preestablished treatment regimens was recommended, unforeseen modifications were observed related to personal protective equipment (PPE) shortages and operating room limitations that may not be anticipated.

Enhanced precautions, including necessary PPE utilization, help mitigate the risk of airborne transmission of SARS-CoV-2 during head and neck examinations and interventions. Restrictions in aerosol-generating procedures in multiple practice settings resulted in a significant reduction in outpatient clinic volume by 62% compared to the prior year. Telemedicine evaluations have been the primary form of oncologic surveillance and postoperative examinations, if possible. In the setting of necessary in-person visits, N95 respirators or powered air-purifying respirators were used to limit risk of transmission during aerosol-generating procedures. Furthermore, in-office endoscopic examinations were limited to only necessary diagnostic or surveillance procedures that would influence a decision on treatment consistent with guideline recommendations for patients with head and neck cancer.^18^ In the setting of these restrictions, the findings of the study identified oncologic care continued with limited modifications.

Prioritization of cancer care is in line with guidance from the American College of Surgeons, which defined mucosal cancers of the upper aerodigestive tract (UAT) as high-acuity cases in which treatment should not be delayed.^19^ Compared to the previous year, there were a greater number of new cancer presentations and a greater number of total cases presented during the tumor conference. While there were overall reductions in the number of total cases performed and patients seen in the outpatient clinic, there was a greater proportion of new cancer consultations and oncologic surgeries compared to the prior year to suggest there was a prioritization of oncologic care. The types of consultations and procedures that were eliminated included elective procedures for benign neoplasms and nonemergent reconstructive surgeries.

Treatment modifications were rare and limited to only 10 of 117 patients (8.4%). There were no treatment recommendations that deviated from standard-of-care guidelines. Modifications occurred early in the institutional and state response to the pandemic, as there was greater uncertainty during this time period regarding PPE, availability of virus testing, and levels of risk based on specific exposures. As these factors became more predictable, there were fewer treatment modifications related to delays in care. For example, there were 7 modifications in the first month of the study period and 3 modifications in the last 2 months. Although there were few modifications overall, some general trends were noted. The most common modification for surgical management was a delay related to operating room safety or delays in COVID-19 testing. While most modifications occurred due to institutional response or patient preferences, some modifications were recommended by the MDTB. These modifications related primarily to some patients with human papillomavirus (HPV)–associated oropharyngeal cancer when there was clinical equipoise between surgical or nonsurgical management. In these instances, nonsurgical management was recommended to avoid longer hospital stays and the need for aerosol-generating procedures.

Although many groups have predicted substantial treatment modifications and delays in access to care, there remains limited evidence of the observed impact of oncologic care access for patients with head and neck cancer. There has been literature offering consensus-based recommendations, survey findings, or opinion regarding the appropriate triage of patients with head and neck cancer. Bowman et al^20^ predicted a surge in patients with head and neck cancer after COVID-19 recovery. They cited concerns of contracting the virus, limitations of testing, and local and state restrictions as reasons new cancer patients would delay seeking care. A complementary study published by Brody et al^14^ reported survey results from a large group of head and neck surgeons. There was a wide range of responses, but respondents were more likely to consider nonsurgical management and to accept delays in care in the setting of the pandemic.

A recent publication by Kiong et al^21^ offered the first reported changes in tumor conference and clinic volumes in the setting of the ongoing pandemic. The study from the MD Anderson Cancer Center reported a 47% reduction in outpatient visits and a 47% decline in operative volume compared to a 61% and 27% reduction, respectively, in the current series. In contrast to their experience, we saw no significant difference in the number of cases presented at the MDTB. However, there was a similarly low rate of treatment modifications between the MD Anderson Cancer Center experience and our study, 12.0% and 8.4%, respectively.^21^ The unique institutional experience at the MD Anderson Cancer Center, as an independent cancer center, may not reflect national trends as it serves as a primary oncologic hospital. The MD Anderson Cancer Center is a tertiary care center specializing in oncologic care and may not have had the opportunity to delay nonemergent surgeries to facilitate and expedite oncologic care. In contrast, the suspension of elective surgery and operating room block time at UMMC increased operating room availability for urgent surgeries. While the prioritization of oncologic care at UMMC may have led to a low rate of modifications, this is reflective of the institutional experience. While our institution may be similar to others across the country, our findings should be interpreted within the context of the pandemic experienced in our region. While there are similarities in the institutional experiences, the differences highlight the need for tailored approaches in each institution and geographic setting.

### Study Limitations

Our study has several limitations. There was a short follow-up period as well as the lack of multiple years of historical data for comparison for our MDTB patients. An unanticipated finding during the study period is the inverse relationship observed with the rise in MDTB presentations and the concurrent decline in clinical and surgical volume. This observation may be attributable to the MDTB virtual format that allowed for remote access, resulting in more cases being presented from faculty in various practice settings. In contrast, the lower reported MDTB rates in 2019 are potentially related to distance barriers and delays during in-person meetings. The ability of the virtual format to increase participation in the conference may offer a more robust multidisciplinary participation compared to prior in-person meetings. Furthermore, a portion of the decrease in outpatient clinic visits may be accounted for by telemedicine visits, but these primarily served to replace routine follow-up visits rather than initial consultations. Our results reflect the patterns of care at a single institution, and our data may reflect a regional impact of the COVID-19 pandemic. Our ability to capture modifications and delays in care is limited by the characteristics of patients who present for care at our institution, and therefore our findings may underestimate the true impact of the pandemic. Institutions in various regions may have different state-mandated restrictions and institutional resources that make each experience unique. Despite these limitations, the study emphasizes the prioritization of care for patients with head and neck cancer as well as the utility of reviewing the impacts of the pandemic.

## Conclusion

The COVID-19 pandemic has resulted in changes in practice patterns for oncologic care. The transition to a virtual tumor board format resulted in an increase in new cancer presentations for head and neck cancer, while in-person clinical care, including outpatient visits and operative procedures, was reduced compared to historical data. Despite overall reduction in clinical volume, the increased proportion of oncologic consultation and cases demonstrates that prioritization for head and neck cancer in both settings. As the COVID-19 pandemic continues, with possibilities of additional peaks in case volumes, institutions will need to continue to use resources to streamline care for oncologic patients. They will need to rely on technology, optimal use of personal protective equipment, and adaptation while emphasizing standard of care to achieve the best outcomes for patients with head and neck cancer.
